# Implementing Connect ME: Partnering With Meals on Wheels Agencies to Improve Social Connectedness

**DOI:** 10.1093/geroni/igaf122.1575

**Published:** 2025-12-31

**Authors:** Renee Pepin

**Affiliations:** Geisel School of Medicine at Dartmouth, Lebanon, New Hampshire, United States

## Abstract

**Aim:**

To test the reach and fit of Brief Behavioral Activation for Improving Social Connectedness, an intervention to increase social connectedness among homebound older adults living in rural Maine.

**Methods:**

We partnered with two Maine Area Agencies on Aging (AAA) that deliver Meals on Wheels (MoW) to conduct a study examining the implementation of Connect ME, a program to improve social connectedness which has two components 1) systematic loneliness screening for all clients during routine MoW assessments and 2) Brief Behavioral Activation for Improving Social Connectedness, a 6-session intervention, which was embedded in one partner AAA and delivered by their AAA staff. We assembled a blended research team, which included academic researchers and AAA staff. To test reach, we collected data on screening and recruitment. To test fit, we conducted qualitative interviews with AAA staff.

**Results:**

Over 2,000 MoW clients were screened, with 18.8% screened as lonely. Of those, 21% opted to participate in additional eligibility assessment for the Behavioral Activation component. The blended research team identified opportunities to improve procedures in real time. AAA staff reported that screening for loneliness and embedding Brief Behavioral Activation for Improving Social Connectedness were a good fit for AAA organizations. Staff identified key organizational factors that contributed to screening and intervention delivery success. Staff identified significant barriers regarding engaging MoW clients in the Behavioral Activation intervention along with potential solutions for future implementation.

**Implications:**

Key findings of organizational factors related to success and challenges can be applied to future Aging Service implementation.

